# Environmental factors affect the distribution of two *Epichloë* fungal endophyte species inhabiting a common host grove bluegrass (*Poa alsodes*)

**DOI:** 10.1002/ece3.5241

**Published:** 2019-05-26

**Authors:** Tatsiana Shymanovich, Stanley H. Faeth

**Affiliations:** ^1^ Biology Department University of North Carolina at Greensboro Greensboro North Carolina

**Keywords:** ecological factors, endophyte distributions, endophyte‐host compatibility, grass populations, host plant growth, infection benefits, southeastern North America

## Abstract

**Aim:**

The endophyte *Epichloë alsodes*, with known insecticidal properties, is found in a majority of *Poa alsodes* populations across a latitudinal gradient from North Carolina to New York. A second endophyte, *E. schardlii* var. *pennsylvanica*, with known insect‐deterring effects, is limited to a few populations in Pennsylvania. We explored whether such disparate differences in distributions could be explained by selection from biotic and abiotic environmental factors.

**Location:**

Along the Appalachian Mountains from North Carolina to New York, USA.

**Taxon:**

Fungi.

**Methods:**

Studied correlations of infection frequencies with abiotic and biotic environmental factors. Checked endophyte vertical transmission rates and effects on overwintering survival. With artificial inoculations for two host populations with two isolates per endophyte species, tested endophyte–host compatibility. Studied effects of isolates on host performances in greenhouse experiment with four water‐nutrients treatments.

**Results:**

Correlation analysis revealed positive associations of *E. alsodes* frequency with July Max temperatures, July precipitation, and soil nitrogen and phosphorous and negative associations with insect damage and soil magnesium and potassium. Plants infected with *E. alsodes* had increased overwintering survival compared to plants infected with *E. schardlii* or uninfected (E−) plants. Artificial inoculations indicated that *E. alsodes* had better compatibility with a variety of host genotypes than did *E. schardlii*. The experiment with reciprocally inoculated plants grown under different treatments revealed a complexity of interactions among hosts, endophyte species, isolate within species, host plant origin, and environmental factors. Neither of the endophyte species increased plant biomass, but some of the isolates within each species had other effects on plant growth such as increased root:shoot ratio, number of tillers, and changes in plant height that might affect host fitness.

**Main conclusion:**

In the absence of clear and consistent effects of the endophytes on host growth, the differences in endophyte‐mediated protection against herbivores may be the key factor determining distribution differences of the two endophyte species.

## 1 INTRODUCTION

Plant microbial symbionts, such as various groups of fungi and bacteria, play an important role in plant stress resistance against various abiotic and biotic selective pressures (Johnson, Graham, & Smith, 1997; Rodriguez, White, Arnold, & Redman, 2009; Rosenblueth & Martínez‐Romero, 2006; Schulz, 2006). For example, when resources such as soil nutrients are limiting, host plants may partner with microorganisms to increase nutrient uptake. Nitrogen‐fixing *Rhizobium* bacteria are well known for increasing nitrogen availability to legumes, and ectomycorrhizal and arbuscular mycorrhizal fungi increase nutrients and water uptake in many vascular plant species (Bordeleau & Prévost, 1994; Entry, Rygiewicz, Watrud, & Donnelly, 2002; Smith & Read, 2010). These symbioses with beneficial microbes may be an essential mechanism for increasing plant fitness and thus expanding host plant niche and distribution into habitats where the host plant could not otherwise persist (Bordeleau & Prévost, 1994; Friesen et al., 2011; Kazenel et al., 2015; Mapfumo, Mtambanengwe, Giller, & Mpepereki, 2005; Reynolds, Packer, Bever, & Clay, 2003).

One group of plant symbionts, *Epichloë* species, systemic endophytic fungi of cool season grasses, has been shown to mitigate the effects of environmental stress such as drought and nutrient deficiencies as well as anthropomorphic stresses such as elevated CO_2_ associated with climate change and resisting invasive species (Brosi et al., 2011; Compant, Heijden, & Sessitsch, 2010; Craig et al., 2011; Malinowski & Belesky, 2000). Moreover, these fungi may produce alkaloid compounds that have toxic or deterrent effects on various herbivores, thus reducing environmental stress from insect herbivory and vertebrate grazing (Brosi et al., 2011; Cheplick & Faeth, 2009; Compant et al., 2010; Craig et al., 2011; Hunt, Rasmussen, Newton, Parsons, & Newman, 2005; Malinowski & Belesky, 2000; Schardl, Balestrini, Florea, Zhang, & Scott, 2009). The mode of transmission of *Epichloë* endophytes varies, with some species transmitted either vertically (via hyphae growing into seeds) or horizontally (by forming stromata and causing disease symptoms) or via both modes depending on the environment (Clay & Schardl, 2002). *Epichloë* endophytes that are thought to be strictly vertically (maternally) transmitted are considered more strongly mutualistic because host plant and endophyte reproduction, and hence fitness, are closely linked (Cheplick & Faeth, 2009; Clay & Schardl, 2002).

However, hosting the endophyte, whether it is vertically or horizontally transmitted, entails metabolic and nutritional costs for the host grass. Alkaloid biosynthesis is metabolically costly and also requires nitrogen, which is often limiting for plant growth (Faeth & Fagan, 2002). In resource‐poor environments, hosting an endophyte may be too costly and outweigh the associated benefits (Ahlholm, Helander, Lehtimäki, Wäli, & Saikkonen, 2002; Cheplick, 2007; Faeth & Sullivan, 2003; Rasmussen, Parsons, Fraser, Xue, & Newman, 2008). Thus, beneficial effects of harboring an *Epichloë* species are not fixed, and host–endophyte interactions may range from mutualistic to parasitic depending on the mode of transmission (vertical vs. horizontal), genetic compatibility of the host, and endophyte species or strain, and abiotic and biotic ecological factors (e.g., Cheplick & Faeth, 2009; Faeth, 2002; Saikkonen, Wäli, & Helander, 2010; Schardl et al., 2009). For example, some studies show no effect of *Epichloë* infection on drought stress tolerance of native grass hosts, or even reduced resistance to stress, depending on endophyte species and the environmental conditions (Cheplick, Perera, & Koulouris, 2000; Jia, Shymanovich, Gao, & Faeth, 2015; Morse, Faeth, & Day, 2007).

Generally, little is known about the effects of endophytes on their hosts across natural populations from different environments (e.g., Cheplick & Faeth, 2009; Hamilton, Faeth, & Dowling, 2009; Novas, Collantes, & Cabral, 2007; Wei et al., 2007). Basic knowledge of the variation in endophyte species and strains and their frequencies over a geographic range of environmental conditions may provide insights into the long‐term nature of the interactions of endophytes and their hosts. Genetics of host plants also varies over the range of a grass species and may interact with variation in endophyte species or strain to affect persistence of the plant–endophyte symbiota. Indeed, host and endophyte genotypic combinations, especially in maternally transmitted endophytes, may have co‐evolved with each other to increase fitness, and thus may be adapted to local environmental conditions (Cheplick & Faeth, 2009; Oberhofer, Gusewell, & Leuchtmann, 2014; Saikkonen et al., 2010). Correlational studies may provide insight into what environmental factors are associated with different endophyte species or strains within a common host grass.

**Figure 1:**
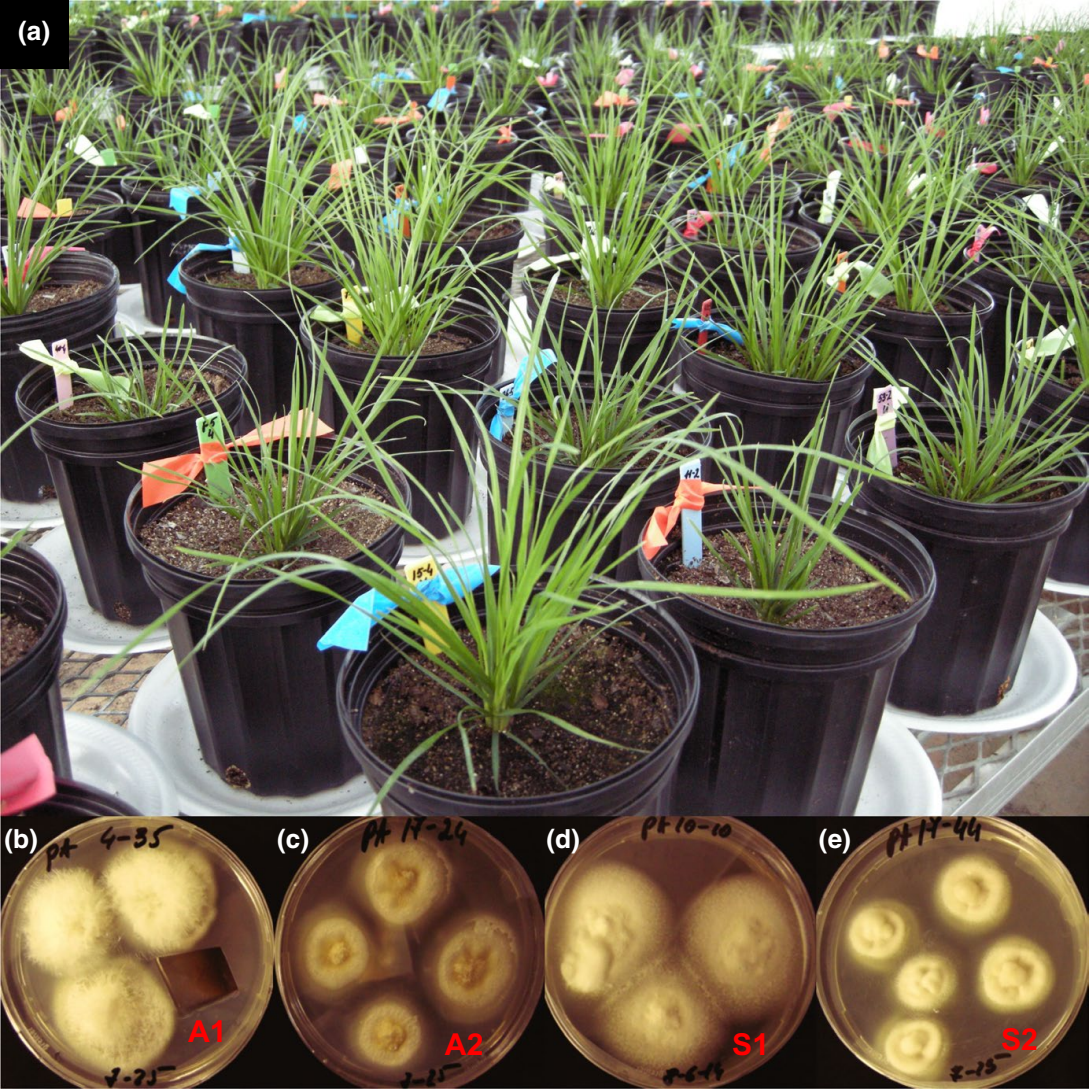
(a) *Poa alsodes* plants inoculates with *Epichloë alsodes*, A1 and A2 isolates (b, c), and *Epichloë schardlii* var. *pennsylvanica*, S1 and S2 isolates (d, e) from the greenhouse experiment with different water‐nutrient treatments

In one native grass, *Poa alsodes*, common in eastern North America, *Epichloë* infection frequencies vary across a latitudinal gradient (Shymanovich et al., 2017). Furthermore, two endophyte species, *E. alsodes* and *E. schardlii* var. *pennsylvanica* infect this grass (Figure 1). However, the two *Epichloë* species dramatically differ in their distributions, alkaloid genetic profiles, and the different strategies used to defend against insect herbivory (Shymanovich et al., 2017; Shymanovich, Musso, Cech, & Faeth, 2019). *E. alsodes* produces the toxic insecticidal alkaloid, *N*‐acetylnorloline (NANL), while *E. schardlii* var. *pennsylvanica* has insect‐deterring properties due to unidentified allelochemicals or some other mechanism. *E. alsodes*, an interspecific hybrid, was observed in 23 out of 24 populations studied across a latitudinal gradient of about 1,200 km along the Appalachian Mountains in eastern North America. In contrast, *E. schardlii* var. *pennsylvanica*, an intraspecific hybrid of two *E. typhina* strains, showed a restrictive distribution and was observed only in a few populations in Pennsylvania.

From 34 described *Epichloë* species and several subspecies, 19 are interspecific hybrids, one is intraspecific hybrid, and 21 are nonhybrids (Ghimire, Rudgers, Charlton, Young, & Craven, 2011; Leuchtmann, Bacon, Schardl, White, & Tadych, 2014; Schardl & Craven, 2003). Interspecific hybrids are thought to have added genetic variation that adapts them to a wider range of environments (Schardl & Craven, 2003). We found only one *P. alsodes* population where *E. schardlii* var. *pennsylvanica* was the sole endophyte infection (74% infection rate), and four other populations where it was mixed with the more common endophyte, *E. alsodes* (Shymanovich et al., 2017). Total *Epichloë* infection rates, mainly because of *E. alsodes*, were 90%–100% in the majority of populations. One *P. alsodes* population, however, had an *E. alsodes* infection rate of only 26%. The intraspecific hybrid, *E. schardlii*, was initially described from the host *Cinna arundinacea*, and the role of this endophyte in host growth has not yet been explored (Ghimire et al., 2011; Leuchtmann et al., 2014). Thus, this *P. alsodes* host grass system is unique because it is the only grass host species known to date where an interspecific or intraspecific hybrid *Epichloë* species co‐occur.

Selection by the biotic and abiotic environment largely controls whether the costs of harboring *Epichloë* endophytes outweigh the benefits or vice versa, and the outcomes of this selection over time may be reflected in endophyte distributions and frequencies across the populations. Correlation with environmental factors can point to possible factors that may determine the distribution and relative frequency of the endophyte species. However, the assumption that higher relative frequencies of an endophyte species reflect greater benefits may be misleading because other factors such as differences in rate of endophyte transmission (Afkhami & Rudgers, 2008; Sneck, Rudgers, Young, & Miller, 2017), timing of species origin or host–endophyte associations, meta‐population or meta‐community dynamics, or differences in dispersal may affect frequencies (Faeth & Sullivan, 2003; Saikkonen, Faeth, Helander, & Sullivan, 1998; Saikkonen, Lehtonen, Helander, Koricheva, & Faeth, 2006; Saikkonen, Wali, Helander, & Faeth, 2004). For example, difference in the distributions and relative frequency of the two endophytes could be explained by selection via ecological factors or simply by more recent timing of origin of *P. alsodes*–*E. schardlii* var. *pennsylvanica* host–endophyte association or host jump of *E. schardlii* from *C. arundinacea* to *P. alsodes* in Pennsylvania. Experimental studies where endophyte species, endophyte and plant genotypes, and key environmental factors are controlled and host plant performance is measured, can further assist in determining if ecological factors in conjunction with plant and endophyte genotype can explain differences in endophyte distribution (Jia, Oberhofer, Shymanovich, & Faeth, 2016; Jia et al., 2015; Oberhofer et al., 2014; Vandegrift et al., 2015).

We hypothesized that key environmental factors affect the presence and frequency of *Epichloë* endophyte species in natural populations across a latitudinal gradient. First, we explored if specific abiotic or biotic factors in natural populations of *P. alsodes* are associated with *E. alsodes* infection frequencies across a latitudinal gradient. Similar multiple regression analyses for *E. alsodes* and *E. schardlii* var. *pennsylvanica* were performed for the Pennsylvanian region, the only region where both endophytes co‐occur. To further address what environmental factors may be related to the expansive *E. alsodes* versus restricted *E. schardlii* var. *pennsylvanica* endophyte distribution across our latitudinal gradient, we examined the vertical transmission rates of the two endophytes, and we compared overwintering plant survival for plants infected with either *E. alsodes* or *E. schardlii* var. *pennsylvanica*. We also experimentally tested the compatibility of the endophyte–plant association by experimentally inoculating the residential or alien isolates of the two species into uninfected seedlings from two plant populations. Finally, we tested how infection with a specific endophyte isolate of each species affected plant growth under controlled water and nutrient availability, two key factors for plant growth and survival.

## 2 MATERIALS AND METHODS

### 2.1 Plant host

*Poa alsodes* A. Gray (common name, grove bluegrass), family Poaceae, is a perennial woodland grass species. *P. alsodes* is distributed in eastern North America from Canada to South Carolina, USA. In the southern part of its range, it is restricted to mountainous areas and becomes more widespread in northern regions. Flowering occurs in spring, and plants are mainly out‐crossing via wind pollination, but self‐fertilization is also possible. *P. alsodes* has not been used in agriculture (Hill, 2007).

### 2.2 Endophyte species

The widespread and common endophyte inhabiting *P. alsodes* is *E. alsodes*, which is an interspecific hybrid of *E. typhina* subsp. *poae* and *E. amarillans*. This species has two mating type idiomorphs, *MTA* and *MTB*, and genes for production of *N*‐acetylnorloline, a loline alkaloid. Genes for ergot alkaloids and peramine biosynthetic pathways are not functional (Shymanovich et al., 2017). The less common and more range restrictive endophyte inhabiting *P. alsodes*, *E. schardlii* var. *pennsylvanica*, is closely related to, and most likely is synonymous with, *E. schardlii*, which was described previously from *Cinna arundinacea* hosts (Ghimire et al., 2011; Shymanovich et al., 2017). For simplicity and clarity, we use the *E. schardlii* name for this endophyte here. This endophyte is an intraspecific hybrid of two strains of *E. typhina* subsp. *poae*. This species has the *MTB* idiomorph and the peramine alkaloid gene. However, based on chemical analyses, peramine is not produced (Shymanovich et al., 2017). Both endophytes, like most hybrid *Epichloë* species, appear to be strictly vertically transmitted by hyphae growing into seeds and no stromata have been observed on *P. alsodes* in nature.

### 2.3 Correlations of infection frequencies with abiotic and biotic environmental factors

We determined whether *Epichloë* species frequencies in the natural *P. alsodes* populations are associated with key abiotic environmental factors, including temperature, precipitation, soil nutrients, and a key biotic environmental factor, insect herbivory pressure. Frequencies of *Epichloë* infections of each species from natural populations were determined from field collections in 2012–2014 and reported in Shymanovich et al. (2017). Grass populations were identified by US state and the number of the collection. In that study, infection frequencies were detected from 50 individual plants sampled from a patch or patches within each population. Soil samples collected from each population were analyzed for percent organic matter, estimated nitrogen release, available phosphorus, exchangeable potassium, magnesium, calcium, and soil pH by A&L Eastern Laboratories, Richmond, VA. Usually, soil samples were combined from all patches within each population. However, for two populations (PA‐18 and PA‐19), soil samples were analyzed separately for each patch within the populations. For these two populations, infection frequencies were determined separately for each patch. Monthly temperature and precipitation averages, such as July Max temperature, January Min temperature, July precipitation, and annual precipitation were obtained from http://www.weather.com/weather/wxclimatology/monthly for each State Park or for the nearest town for each population. For population NC‐2 located near Waterville, NC, data from 1948 to 2014 were obtained from the town weather station. For population NC‐4 located in remote area in the Great Smoky Mountains National Park, data were obtained from http://www.ncdc.noaa.gov/data-access/land-based-station-data database for the nearest climatology station Newfound Gap, TN, located at a similar elevation. However, these data were available only starting in 2012.

Insect herbivory pressure for each population was estimated from aboveground leaf material collected in 2012–2014. Estimates were based on all tillers for small plants (1–10 tillers) and on 10 random tillers per plant for larger plants (>10 tillers). First, mean percent of plant area damaged was estimated for each of 50 plants per population when plant material was available using the

$$\text{formula:}\;\%\;\text{Plant}\;\text{Area}\;\text{Damaged}=\frac{\#\;\text{Damaged}\;\text{Leaves}*\left(\%\;\text{Area}\;\text{Score}/100\right)*100\%}{\#\;\text{Total}\;\text{Leaves}},$$

where area scores are 0%, <5%, <10%, <25%, <50% of leaf area damaged. Second, mean percent of plant area damaged was estimated for each population. Insect herbivore pressure was presented as mean percent of plant area damaged for each population.

From all latitudinal collection data (Table S1) and separately for Pennsylvania populations, we correlated *E. alsodes* infection frequency and the environmental factors. From only Pennsylvania populations, we correlated *E. schardlii* var. *pennsylvanica* infection frequency because this endophyte was not found in the other regions that we sampled.

### 2.4 Vertical infection transmission rates

Transmission rates were estimated for each endophyte species in each population because observed population infection frequencies may depend on the effectiveness of vertical transmission, and transmission efficiency may be affected by environmental factors (Hill & Roach, 2009; Rolston, Hare, Moore, & Christensen, 1986; Siegel, Latch, & Johnson, 1985). For example, imperfect transmission (failure of hyphae to grow into seed ovaries or loss of endophyte viability in plants or seeds due to high temperatures), has been used to explain variation in endophyte frequencies in nature (Afkhami & Rudgers, 2008; Liu, Nagabhyru, & Schardl, 2017; Ravel, Michalakis, & Charmet, 1997). To determine transmission rates, infection status of about 24 (depending on availability) seeds from each of three infected mother plants per population was determined with immunoblot assay (Phytoscreen Immunoblot Kit #ENDO7971 Seed; Agrostics, Watkinsville, GA, USA). Mean transmission rate for each population was calculated from the three mother plants for each *Epichloë* species.

### 2.5 Overwintering study

Four‐month‐old *P. alsodes* plants growing in 300 ml^3^ pots in potting mix were clipped periodically during a 20‐day period (leaves were used for insect experiments). These plants were then placed outside the research greenhouse located in Greensboro, NC, on 20 December 2014. All these plants were grown from seeds collected from five natural populations in Pennsylvania 2012–2013 and tested for endophyte infections: *E. alsodes* (35 plants); *E. schardlii* var. *pennsylvanica* (51 plants); and uninfected (38 plants). Plant survival was evaluated after four months on 17 April 2015. In general, Greensboro climate is expected to be warmer than the climate in the NC mountains, but the winter of 2014–2015 was colder than usual. During this four‐month period, day low temperatures were below freezing for 52 days, and on two days, lowest day temperatures recorded was −14.4°C (weather data from http://www.accuweather.com/en/us/greensboro-nc/27401 ).

### 2.6 Inoculations to test endophyte–host compatibility

To test for difference in endophyte–host compatibility for the two endophyte species, different isolates of each species, host plants from different populations, and reciprocal inoculations with endophytes were used. Inoculation success should be positively associated with endophyte species–host plant compatibility (Latchs & Christensen, 1985; Oberhofer et al., 2014). To control for the plant population effects, naturally uninfected seeds (collected in 2012–2013) from the two widely separated *P. alsodes* populations were used (Table S1) (modified from Shymanovich et al., 2017). One population is located at the southern limit of *P. alsodes’* distributional range in North Carolina (NC). This population is found at a high elevation with high precipitation and relatively low summer temperatures. In this NC population, only one endophyte, *E. alsodes*, was observed at relatively low infection frequency (26%). The second, northern *P. alsodes* population is in Pennsylvania (PA), where the two endophyte species co‐occur. However, because of the lower elevation of this population, summer temperatures are higher and precipitation is lower compared to the NC population. To incorporate endophyte variation within species, two mycelial isolates for each species were obtained from different populations for the artificial inoculations (Table S2).

For the *E. alsodes* endophyte, one isolate (A1) was from the NC population, and the second (A2) was from the PA population. For *E. schardlii* var. *pennsylvanica*, one isolate (S1) was from a different population in Pennsylvania where only this endophyte species was present, and the second (S2) was from the PA population described above where the two endophyte species co‐occur. In this experiment, due to time and budget limitations, we were unable to take into account possible genetic variation within a given population of plants between naturally uninfected and plants infected with a specific endophyte. The latter requires removing the endophyte and growing these plants at least for one year in a natural environment to produce seeds. Therefore, for the NC seedlings, we attempted to introduce A1, a residential isolate, and A2, S1, and S2, three alien isolates. For the PA seedlings, we attempted to introduce A2 and S2, residential isolates, and A1 and S1, alien isolates (Figure 1).

Two endophyte inoculation techniques were employed: with and without seedling puncturing (Figure S1). On 18 September 2014, for each isolate, 17 potato dextrose agar plates were inoculated by pouring on to their surface a suspension of fresh fungal mycelium stirred in sterile water by a pestle. Plates were kept in the dark at 24°C. For each population, seeds from four naturally uninfected mother pants were used. Infection status of each mother plant was verified by PCR (Shymanovich et al., 2017). About 2,300–2400 surface sterilized seeds (1 min 70% ethanol, 4 min 4% sodium hypochlorite, 1 min 70% ethanol, 1 min sterile water), from each population, were split into four isolate groups, evenly placed on ten‐day‐old cultures, and kept in the dark, 24°C for the next 10 days (similarly to Tadych, Bergen, & White, 2014). Plates were then transferred into an Adaptis A1000 (Conviron, Canada) growth chamber set at 25°C and 16/8 light/dark schedule (Figure S2). When germination began during three weeks, each 3–6 mm seedling was punctured under laminar flow with sterile BD PrecisionGlide™ 0.4 × 13 mm needle into a hypocotyl near the seed coat, and a small portion of surrounding mycelium was introduced into a wound using a microscope at 400× and light source (puncturing treatment) as described in Latchs and Christensen (1985) and Oberhofer et al. (2014). Plates were checked for germination every 2–3 days, and newly processed seedlings were marked on the lid (Figure S2). After 7–8 days, inoculated seedlings were individually removed from the agar and planted in 50 ml pots with potting soil (Metro mix‐360, Sun Gro Horticulture Canada Ltd) in greenhouse. *P. alsodes* is a woodland grass and needs reduced light conditions. Therefore, two layers of sunscreen mesh were placed on the greenhouse, and plants received 60%–65% of natural light [measured by Lutron LX‐105 (Lutron Electronics)]. Similar light reduction levels were applied in the other experiments with this grass (Davitt, Stansberry, & Rudgers, 2010). Day/night temperatures were set at 25°C/20°C.

In the mycelia treatment, seedlings that emerged on mycelial plates after three weeks were planted into soil. When surviving seedlings developed several leaves, their infection status was checked from a single leaf sheath per plant with an immunoblot assay (Phytoscreen Immunoblot Kit #ENDO7973 Tiller; Agrostics). All seedlings with positive results for endophyte infection were repotted into 300 ml^3^ pots. A few NC and PA seedlings that tested negative were also repotted and used as uninfected controls. When plants developed several tillers, one tiller was removed to confirm infection status and to identify the *Epichloë* species (*E. alsodes* or *E. schardlii* var. *pennsylvanica*) with PCR genotyping method described in Shymanovich et al. (2017). Inoculation success was evaluated for each plant‐isolate combination as number of positively infected seedlings/total number of survived seedlings for each inoculation procedure (puncturing and mycelia) separately × 100%. Total inoculation success was calculated as total number of positive seedlings/total number of seedlings survived × 100%.

### 2.7 Effects of endophytes on plant performances

To test the effects of endophyte species and plant genotype on plant performance, we used infected seedlings from the inoculations and negative controls (seedlings that were inoculated but remained negative) from NC and PA populations (NC‐E‐ and PA‐E‐). For *E. alsodes* infected plants, we had all the expected combinations: NC‐A1, NC‐A2, PA‐A1, and PA‐A2. For *E. schardlii* var*. pennsylvanica* infected plants, we only had sufficient numbers for NC‐S1 and NC‐S2. Due to poor inoculation success for PA‐S1 and PA‐S2 groups, they were excluded from this experiment. Therefore, we were unable to compare the effects of *E. schardlii* var. *pennsylvanica* infections on plants from the two populations.

Plants with verified infections (n_NC‐A1_ = 12, n_NC‐A2_ = 13, n_NC‐S1_ = 15, n_NC‐S2_ = 6, n_NC‐E‐_ = 10, _nPA‐A1_ = 3, n_PA‐A2 = _7, n_PA‐E‐_ = 8) were maintained in the greenhouse until February 2015. To increase replicates, plants were divided into separate tillers and each tiller potted in two‐liter pots, and then clipped to the same height. Fifty clones were produced for each remaining seed–endophyte combinations, except PA‐A1, which had 42 clones. To test how key environmental factors, such as water and nutrient availability, affect the growth of each symbiotum, we subjected 11–13 plants per symbiotum to one of four randomly assigned treatments (high water/high nutrients (HWHN), high water/low nutrients (HWLN), low water/high nutrients (LWHN), low water/low nutrients (LWLN)) beginning on 1 March 2015. High water treatment plants received about 2× more water than the low water groups twice a week. Water amounts were increased as plants grew during the experiment and soil moisture measurements (measured three times during the experiment from 21 random plants from each treatment group with Dr. Meter^R^ Moisture Sensor, China) confirmed the targeted “Moist” versus “Dry” moisture levels differences in treatments. High‐nutrient groups received 1.48 g/L [20:20: 20 (N: P: K), with micronutrients] (Southern Agricultural Insecticides, Inc.) twice a month. Low nutrient groups did not receive any fertilization during the experiment. Similar treatments were shown to be effective in the other studies (Jia et al., 2015; Saari & Faeth, 2012) to achieve significant differences in plant growth. Plant positions were rotated every 10 days to minimize any microclimatic differences within the greenhouse.

The experiment continued for 97 days after treatments began. On 5 June 2015, plant height and number of tillers were recorded, and then plants were harvested. Aboveground and belowground biomass was separated, dried (three days at 65°C in a drying oven), and shoot and root dry biomass were determined, and root: shoot ratio, as a measure of plant resource allocation, was calculated. A few plants did not survive to the end of the experiment and were excluded from the statistical analyses. Infection status for each plant was confirmed with immunoblot assay (as described above). The infection status of all plants except one (negative instead of positive) was as expected. This plant was excluded from the statistical analyses.

### 2.8 Statistical analyses

Statistical analyses were performed with R i386 3.3.2 software with “*R commander*” package (R Development Core Team, 2008).

#### 2.8.1 Multiple regression analyses

To explore the relationship of endophyte frequencies with environmental factors, we used a multivariate regression analyses of *E. alsodes* infection frequencies with all of the measured environmental factors from the collection of populations across the latitudinal gradient. One population, MI‐20, was removed from analyses because soil data were missing. Two other populations, PA18‐L4 and NY11, were removed later from analyses as outliers based on QQ plots residuals. After a stepwise backward/forward model selection, the best model, based on the lowest BIC score, was determined. For the Pennsylvania populations, where *E. alsodes* and *E. schardlii* var. *pennsylvanica* co‐occur, another multivariate correlation analysis was used for *E. alsodes* and for *E. schardlii* var. *pennsylvanica*. To reduce number of variables, soil calcium and organic matter variables were removed because they were strongly correlated with other variables (pH and nitrogen release, respectively). Based on QQ plots residuals, one outlier, population PA19‐L1, was removed from the both multivariate correlation analyses. The best models for Pennsylvania populations were selected based on the lowest BIC scores using backward/forward model selection.

#### 2.8.2 Overwintering survival

For overwinter survival comparisons, Pearson's Chi‐squared tests were applied for three groups and pairwise combinations. Comparisons of inoculation success were performed with similar Pearson's Chi‐squared tests.

#### 2.8.3 Greenhouse performance experiment

Multi‐way ANOVA models were first used to test for differences among plants with *E. alsodes* isolates and uninfected plants from the two populations with endophyte, plant population, treatment, and their interactions as fixed factors for the following variables: total plant dry biomass, plant height, number of tillers, leaf dry biomass, root dry biomass, and root: shoot ratio. To meet normality assumptions, total plant dry biomass, number of tillers, leaf dry biomass, and root: shoot ratio variables were natural logarithm transformed. We then used multi‐way ANOVA models with endophyte, treatment, and their interaction for each growth parameter (same transformations used) for the NC population with *E. alsodes* and *E. schardlii* var. *pennsylvanica* infected and uninfected plants.

To compare effects of the isolates across all treatments on plants from each population, Tukey HSD tests for multiple comparisons of variable means for each growth parameter were used for the effect of endophyte and treatment on growth parameters of NC and PA population plants separately.

To determine whether genetic background of the uninfected plants from the two populations affected performance, multi‐way ANOVA tests with plant population, treatment and their interaction as fixed factors were performed. In these ANOVAs, only uninfected plants from the populations were considered so that endophyte infection would not be a confounding factor for any differences in plant performance.

To compare the effects of resident versus alien endophyte for *E. alsodes* isolates, multi‐way ANOVA tests with endophyte, treatment, and their interactions were used for only infected A1 and A2 groups for each population separately. The same transformations as above were applied.

To determine the effects of the specific infections within each treatment, one‐way ANOVA comparisons for all variables were used for each plant population with endophyte as a fixed factor. The same transformations as above were used.

## 3 RESULTS

### 3.1 Regression analyses of endophyte infection frequencies with environmental factors

*Epichloë alsodes* infection frequencies across the latitudinal populations of *P. alsodes* were associated positively with July Max temperature, July precipitation, soil organic matter, phosphorous, and pH, and negatively with soil magnesium, potassium, and mean insect damage (best‐fit regression model, *F* = 10.93 on 9 and 9 *df*, *p*‐value 0.0007, *R* ^2^ = 0.83) (Table 1). For the Pennsylvania data set for *E. alsodes*, infection frequencies were positively associated with soil nitrogen and phosphorous and negatively associated with potassium, magnesium, and mean insect damage (*p* = 0.076) (best‐fit regression model, *F*‐statistics 32.07 on 6 and 4 *df*, *p*‐value 0.002, *R* ^2^ = 0.95) (Table 2). For the Pennsylvania data set for *E. schardlii*, soil magnesium, nitrogen, phosphorous and potassium were correlated with infection frequencies (best‐fit model *F*‐statistic 31.53 on 4 and 6 *df*, *p*‐value 0.0004, *R* ^2^ = 0.92). Moreover, the directions of these regression coefficients were opposite than for *E. alsodes* (Table 2).

**Table 1:** Stepwise selected multiple regression model correlation coefficients between infection frequency of *E. alsodes* and abiotic and biotic environmental factors from *Poa alsodes* populations across the latitudinal gradient

Variable	Correlation coefficient	*p*‐value
January min temperature	−4.16	0.11
July max temperature	11.84	0.009
July precipitation	0.69	0.036
Soil magnesium	−0.16	0.002
Mean percent of insect damage	−11.51	0.0002
Soil organic matter	7.88	0.013
Soil pH	12.30	0.01
Soil phosphorous	1.63	0.0002
Soil potassium	−1.38	0.0001

**Table 2:** Summary of multiple regression analyses for *E. alsodes* and *E. schardlii* var. *pennsylvanica* endophyte distributions with abiotic and biotic environmental factors in *Poa alsodes* populations in Pennsylvania

Variable	*E. alsodes*		*E. schardlii* var. *pennsylvanica*	
	Coefficient	*p*‐value	Coefficient	*p*‐value
Magnesium	−0.06	0.03	0.07	0.024
Nitrogen release	3.26	0.0008	−3.39	0.0001
Phosphorous	1.80	0.002	−1.20	0.001
Potassium	−2.23	0.0005	1.62	<0.0001
Mean insect damage	−4.31	0.076	—	—
July precipitation	1.15	0.16	—	—

### 3.2 Endophyte vertical transmission rates

Transmission rates were high for both endophyte species. For *E. alsodes*, 15 populations were estimated at 100%, and two at 98.61% transmission rate from maternal plants to offspring seeds. For *E. schardlii* var. *pennsylvanica*, four populations were estimated at 100% and one at 95.83% transmission rate from maternal plants to seeds (Table S3).

### 3.3 Overwintering survival test

*Poa alsodes* plants from the three groups varied in their survival rates after four winter months (*p* = 0.001, Pearson's Chi‐squared). E− plants and *E. schardlii* var. *pennsylvanica* infected plants had similar survival rates of 37% and 29%, respectively (*p* = 0.4, Pearson's chi‐squared). However, plants with *E. alsodes* endophyte show significantly higher survival (69%) than the E− group plants (*p* = 0.007, Pearson's chi‐squared) and the *E. schardlii* var. *pennsylvanica* infected plants (*p* = 0.0003, Pearson's Chi‐squared).

### 3.4 Reciprocal inoculation success

Seedlings grown from maternal plants originating from NC and PA populations differed in their compatibility with the two endophyte species (Pearson's chi‐squared test for successful inoculations for both species per population vs. survived seedlings, *p* < 0.0001) (Table 3). For the NC population, successful inoculations were achieved for all four mycelia groups. For the PA population, only three groups were successfully inoculated (inoculations with the S2 isolate failed). Moreover, percent of total successful inoculations was higher for all NC plant groups compared to PA plant groups (Table 3). Seedlings from the NC population showed similar compatibility with the A1 (residential) and A2 (alien) endophyte and as well with S1 and S2, *E. schardlii* alien isolates. Differences for four mycelia inoculations in NC population plants were not statistically significant (Pearson's Chi‐squared test, *p* = 0.3). For the NC population, the puncturing procedure was slightly more successful than the mycelia treatment for *E. alsodes* endophyte (Pearson's chi‐squared test, *p* = 0.07). For *E. schardlii*, mycelia treatments were more successful but not statistically so (Pearson's chi‐squared test, *p* = 0.1).

**Table 3:** Inoculation success for surviving seedlings from North Carolina (NC) and Pennsylvania (PA) populations

Seed population	Endophytes/isolates			
	*E*. alsodes, A1	*E*. alsodes, A2	*E. schardlii* var. *pennsylvanica*, S1	*E. schardlii* var*. pennsylvanica*, S2
**NC Total success/total seedlings**	**12/67 (18%) Resident**	**13/67 (19%) Alien**	**15/59 (25%) Alien**	**6/61 (10%) Alien**
NC—success from puncturing	8/30 (27%)	9/35 (26%)	4/22 (18%)	3/40 (7.5%)
NC—success mycelia	4/37 (11%)	4/32 (12.5%)	11/37 (30%)	3/21 (14%)
**PA Total success/total seedlings**	**3/90 (3%) Alien**	**7/91 (8%) Resident/Alien**	**1/114 (0.8%) Alien**	**0/85 (0%) Resident/Alien**
PA—success from puncturing	0/30 (0%)	7/17 (41%)	0/18 (0%)	0/2 (0%)
PA—success mycelia	3/60 (5%)	0/74 (0%)	1/96 (1%)	0/83 (0%)

Numbers in bold show results combined from the two (puncturing and mycelia) inoculation methods.

Successful mycelia inoculations into plants from the PA population were achieved only in three cases. The best success was achieved by puncturing in the A2 group, which is a potential residential endophyte since both endophyte species co‐occur there. Successful inoculations of the alien A1 isolate were achieved only with the mycelium treatment. Inoculations with the S1 alien isolate had very low success rate, with only one plant infected. Surprisingly, there were no successful inoculations with S2, another potential resident endophyte, with either method, but only two plants survived after puncturing.

### 3.5 Performance experiment comparisons for NC and PA plants inoculated with *E. alsodes* isolates and uninfected plants

Analyses of variance for plants inoculated with the two *E. alsodes* isolates and uninfected plants from NC and PA populations revealed that endophyte, plant population, and water–nutrient treatments all affected growth parameters (Table 4). All growth parameters except number of tillers varied among E−, *E. alsodes* (A1) and *E. alsodes* (A2) infected plants (Table 4). The endophyte × population interaction was significant for all variables except plant height. For plant height, the interaction of plant population and treatment was significant. All other interactions were not significant.

**Table 4:** Analysis of variance results for the effects of *E. alsodes* isolates, host population, and drought/nutrient stress on *Poa alsodes*

	*df*	Total biomass		Leaf biomass		Root biomass		Root:shoot		Plant height		Number of tillers	
		*F*	*p*	*F*	*p*	*F*	*p*	*F*	*p*	*F*	*p*	*F*	*p*
Endophyte (E)^a^	2	11.46	**<0.000**	11.21	**<0.000**	13.92	**<0.000**	11.95	**<0.000**	6.79	**0.001**	1.24	0.29
Plant population (P)^b^	1	0.03	0.86	0.00	0.95	1.41	0.24	0.63	0.43	154.31	**<0.000**	68.07	**<0.000**
Treatment (T)^c^	3	170.88	**<0.000**	176.80	**<0.000**	123.46	**<0.000**	48.51	**<0.000**	180.87	**<0.000**	36.33	**<0.000**
E * P	2	3.99	**0.02**	3.69	**0.03**	4.69	**0.01**	3.86	**0.02**	0.42	0.66	5.12	**0.007**
E * T	6	0.74	0.62	0.87	0.52	0.51	0.80	1.24	0.29	0.98	0.44	0.38	0.89
P * T	3	1.49	0.22	1.45	0.23	2.08	0.10	1.01	0.38	7.67	**<0.000**	1.08	0.36
E * P * T	6	0.12	0.99	0.07	1.00	0.36	0.90	0.45	0.85	0.52	0.79	0.69	0.65
Error	254												

Note

*p* < 0.05 are in bold.

a *E. alsodes* isolates A1, A2 inoculated and E−.

b NC and PA populations.

c HWHN, HWLN, LWHN, LWLN treatments.

#### 3.5.1 *E. alsodes* effects

**Figure 2:**
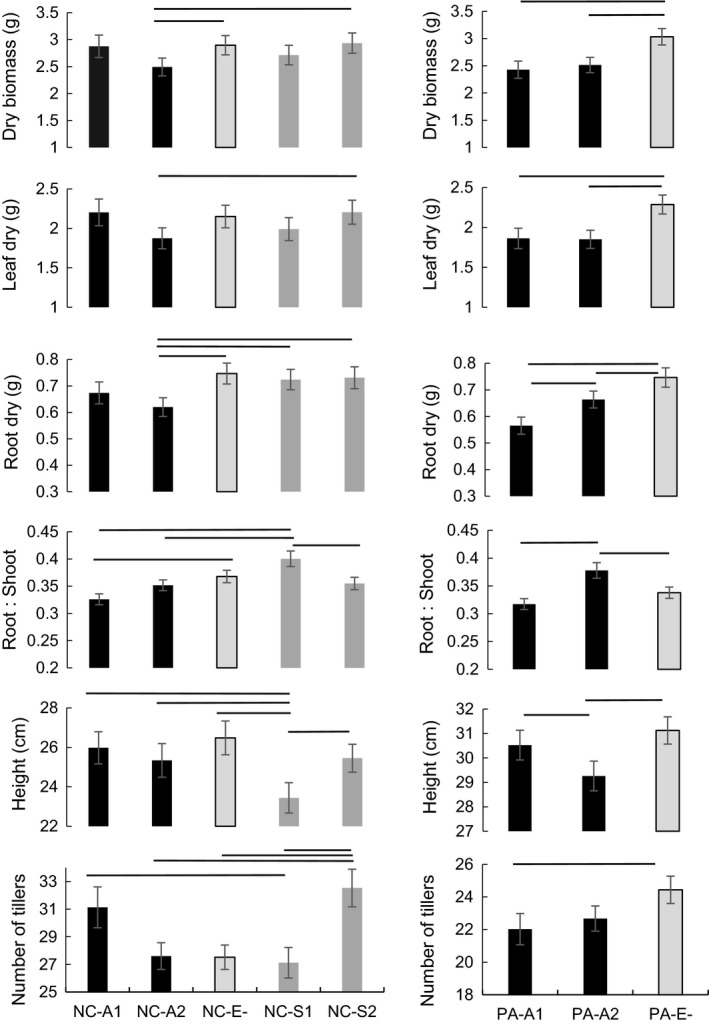
Pairwise comparisons (Means ± *SE*, Tukey HSD) for *Poa alsodes* plants from the greenhouse experiment. Seeds originated from North Carolinian (NC) Great Smoky Mountains National Park (GSM) and Pennsylvanian (PA) Elk State Park (EST) populations. Naturally uninfected seedlings were inoculated with endophytes *Epichloë alsodes* isolates: A1—from GMS, NC, population; A2—from EST, PA, population; *E. schardlii* var. *pennsylvanica* isolates: S1—from Chapman State Park, PA, population; S2—from EST, PA, population; E− stayed uninfected. There were 11–13 plants randomly assigned per treatment for each symbiotum combination. Black horizontal lines designate significant differences (*p* < 0.05) among the same population plants with different infections

Pairwise comparisons of plants originating from NC and PA populations with introduced isolates showed several significant effects of the A1 and A2 isolate infections for some growth parameters but not others (Figure 2). Leaf dry, root dry, and total dry biomass in the inoculated plants were similar or reduced in comparison to the uninfected plants from each population. Total plant biomass was reduced in NC plants inoculated with the A2 *E. alsodes* alien isolate, and for both alien A1 and presumably residential A2 *E. alsodes* isolates in PA plants (Figure 2). However, for NC plants, the effect of inoculating with the A1 or A2 isolate was mainly the reduction of root biomass, while in PA plants, these infections resulted in reduced leaf and root biomass. The two isolates resulted in a range of effects on root: shoot ratio, plant height, and number of tillers when inoculated into plants originating from the same population compared to uninfected plants from these populations. Compared to E− plants from the same population, root: shoot ratio was reduced in NC‐A1 infected plants but remain similar in PA‐A1 plants. Root: shoot ratio was increased by A2 presumably residential endophyte in PA plants and remained the same in NC plants. *E. alsodes* had effects on plant architecture, height versus width (number of tillers) proportions, but only in PA plants. PA‐A2 plants had reduced height and PA‐A1 plants had reduced number of tillers relative to uninfected plants. The two *E. alsodes* isolates changed root dry biomass, root: shoot ratio, and plant height differences only (Figure 2).

#### 3.5.2 Residential versus alien isolate effects within treatments

**Figure 3:**
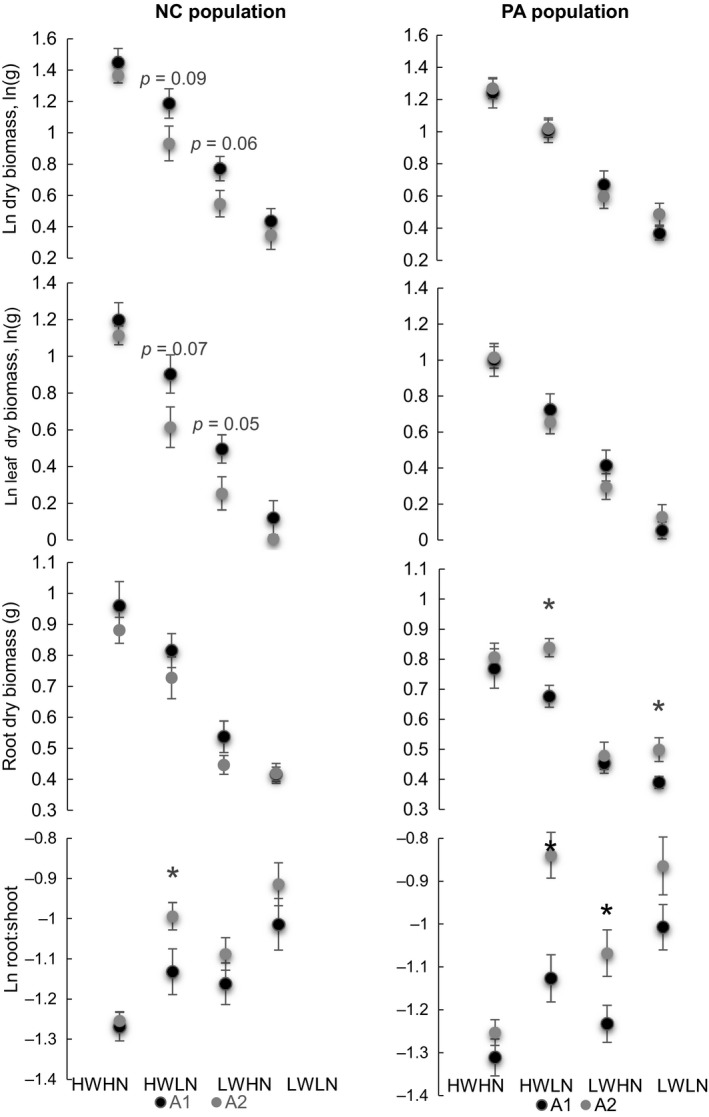
Mean (±SE) of the effects of *E. alsodes* isolates A1 versus A2 on presumably residential versus alien plant hosts from two *Poa alsodes* populations, North Carolina (NC) and Pennsylvania (PA) placed in HWHN, HWLN, LWHN, LWLN treatments. Fungal isolates were artificially inoculated in naturally uninfected seedlings. Residential for the NC population, A1 isolate, represents the only *E. alsodes* infection observed there in *P. alsodes*. A2 isolate represents one of two *Epichloë* species found in the PA population and thus, it may be considered residential for this *P. alsodes* population. There were 11–13 plants randomly assigned per treatment for each symbiotum combination. Asterisks indicate significant differences (*p* < 0.05, one‐way ANOVAs), and for suggestive differences *p*‐values are provided

Two *E. alsodes* isolates, when inoculated into plants from the NC population had different effects on total dry biomass (multi‐way ANOVA, *p* = 0.009), leaf dry biomass (multi‐way ANOVA, *p* = 0.006), root: shoot ratio (multi‐way ANOVA, *p* = 0.01), and number of tillers (multi‐way ANOVA, *p* = 0.01). Treatments were always significant as expected (multi‐way ANOVAs, *p* < 0.001). Mean values comparisons from the models are presented in Figure 3 (data on Ln tillers and height are not shown). Within individual treatments, NC plants infected with residential endophyte (A1) had slightly greater mean total dry and leaf dry biomass in the HWLN, LWHN treatments (Figure 3), and greater tiller number in HWLN treatment (one‐way ANOVA, *p* = 0.02) than plants with alien isolate (A2). However, these plants had reduced root: shoot ratio in HWLN treatment (Figure 3) and reduced tiller number at HWLN treatment (one‐way ANOVA, *p* = 0.02) than NC plants with A2 isolate.

For PA population plants, the fungal isolates affected differently dry root biomass (multi‐way ANOVA, *p* = 0.01), root: shoot ratio (multi‐way ANOVA, *p* < 0.001), and plant height (multi‐way ANOVA, *p* < 0.001). For the plant height model, endophyte × treatment interaction was significant (multi‐way ANOVA, *p* < 0.05). Treatment effects were always significant (multi‐way ANOVAs, *p* < 0.001). When comparing mean values within each treatment, mean dry root biomasses were increased in PA plants inoculated with A2, presumably the residential isolate, in the two low nutrient treatments than in plants with alien (A1) isolate (Figure 3). Also root: shoot ratios were higher in two treatments for A2 plants than for A1 inoculated plants. Height values for presumably residential endophyte (A2) infected plants were lower in the two low nutrients treatments than for plants with alien isolate (A1) (one‐way ANOVAs, *p* = 0.0009, *p* = 0.016).

#### 3.5.3 Plant population

Genetic differences between plants from NC and PA populations affected only plant height and number of tillers (Table 4). Plants from NC and PA populations were similar in other growth parameters, such as total, leaf, and root dry biomass. Similar results were obtained from comparisons of the E− groups only (height *p* < 0.001, tiller number *p* < 0.001, multi‐way ANOVAs). The NC population E− plants tend to be shorter and to have more tillers than E− plants from the PA population (Figure 2). Also, population and treatment interacted to affect plant heights (Table 4). The A1 and A2 isolates affected the height only of PA plants in the HWLN and LWHN treatments (Table 5).

**Table 5:** Summary of significant effects of isolates from *E. alsodes* and *E. schardlii* var. *pennsylvanica* endophyte species on a host plant, *Poa alsodes*, growth parameters for North Carolina (NC) and Pennsylvania (PA) populations under specific treatments

Population/treatment	Ln (total biomass)	Ln (leaf biomass)	Root biomass	Ln (root:shoot)	Height	Ln (number of tillers)
NC/HWHN^a^	—	—	—	—	E− > S1^b^ *p* < 0.05^c^	E− < S2 *p* < 0.05
NC/HWLN	—	—	—	—	—	—
NC/LWHN	—	—	—	A1 < S1 *p* < 0.05	—	—
NC/LWLN	—	—	—	A1 < S1 *p* < 0.05	—	—
PA/HWHN	—	—	—	—	—	—
PA/HWLN	—	—	A1 < A2 = E− *p* < 0.001	A1 < A2 > E− *p* < 0.01	A1 > A2 < E− *p* < 0.01	—
PA/LWHN	A1 = A2 < E− *p* < 0.01	A2 < E− *p* < 0.01	A1 ≤ E− *p* = 0.05	—	A1 = A2 < E− *p* < 0.01	—
PA/LWLN	A1 < E− *p* < 0.05	A1 < E− *p* < 0.05	A1 ≤ E− *p* = 0.05	—	—	—

a Treatments: HWHN—high water high nutrients, LWHN—low water high nutrients, HWLN—high water low nutrients, LWLN—low water low nutrients.

b Infections: E− uninfected; S1, S2‐ infected with *E. schardlii* var. *pennsylvanica* isolates 1 and 2; A1, A2—infected with *E. alsodes* isolates 1 and 2.

c There were 11–13 plants randomly assigned per treatment for each symbiotum combination.

#### 3.5.4 Treatments

**Figure 4:**
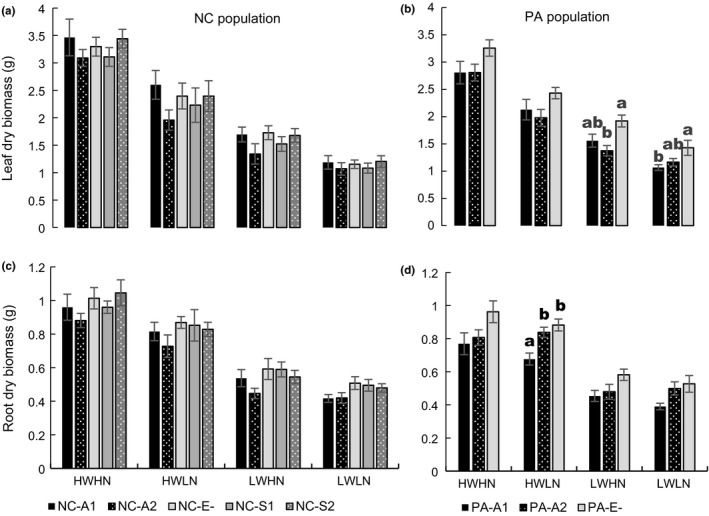
Mean (±*SE*) leaf dry and root dry biomasses for North Carolina (NC) population plants (a, c, respectively) and for Pennsylvania (PA) population plants (b, d, respectively). Naturally uninfected, some NC plants were successfully inoculated with either of two *E. alsodes* isolates (residential A1 and alien A2) or two *E. schardlii* var. *pennsylvanica* isolates (S1 and S2, both alien), or remained uninfected after procedures (E−). Some PA plants were successfully inoculated with either of two *E. alsodes* isolates (alien A1 and presumably residential A2) or remained uninfected after procedures (E−). After infection status check and cloning, plants were randomly assigned into four treatments—HWHN, HWLN, LWHN, LWLN for 97 days. For each symbiotum combination, there were 11–13 plants per treatment. Letters represent statistically significant differences among infection groups within each treatment (one‐way ANOVAs, *p* < 0.05)

As expected, the water‐nutrient treatments strongly affected all growth variables (Table 5). Leaf and root biomass were lower in the LWHN than in HWLN treatment. Plants in the most stressful treatment, LWLN, had the smallest leaf and root biomass (Figure 4). The effects of treatments on plants with a specific infection are discussed below (Table 5).

### 3.6 Effects of *E. alsodes* and *E. schardlii* var. *pennsylvanica* isolates on North Carolina plants

Endophyte infection affected all growth variables for NC plants infected with one of the two isolates for either endophyte species, *E. alsodes* or *E. schardlii* var. *pennsylvanica* (Table 6). As expected, treatments had strong effects on all growth variables. The interaction between endophyte status and treatment was not significant.

**Table 6:** Analysis of variance results for the effects of the isolates of *E. alsodes* and *E. schardlii* var. *pennsylvanica *endophytes on *Poa alsodes* plants from the North Carolina population

	*df*	Total biomass		Leaf biomass		Root biomass		Root: Shoot		Plant height		Number of tillers	
		*F*	*p*	*F*	*p*	*F*	*p*	*F*	*p*	*F*	*p*	*F*	*p*
Endophyte (E)^a^	4	3.10	**0.016**	3.13	**0.016**	3.82	**0.005**	7.68	**<0.000**	5.96	**0.0001**	5.67	**0.0002**
Treatment (T)^b^	3	133.95	**<0.000**	132.39	**<0.000**	106.83	**<0.000**	37.58	**<0.000**	140.28	**<0.000**	32.67	**<0.000**
E * T	12	0.23	1.00	0.23	1.00	0.27	0.99	0.44	0.94	0.36	0.97	0.30	0.99
Error	223												

*p* < 0.05 are in bold.

a *E. alsodes* isolates A1, A2; *E. schardlii* var. *pennsylvanica* isolates S1, S2; E− plants.

b HWHN, HWLN, LWHN, LWLN treatments.

#### 3.6.1 Epichloë schardlii effects

Neither of the *E. schardlii* isolates had significant effects on total, leaf, and root biomass compared to uninfected NC plants (Figure 1). Plants inoculated with the S2 isolate had reduced root: shoot ratio in comparison to plants inoculated with the S1 isolate. The two isolates also had variable effects on plant architecture. Plants inoculated with the S1 isolate had reduced height in comparison to E− plants and plants inoculated with the S2 isolate. S2 infected plants had increased number of tillers when compared to E− plants and S1 infected plants (Figure 2).

#### 3.6.2 Effects of the four isolates

The effects of endophyte infection depended more on the specific isolate than on the *Epichloë* species. Isolates of each endophyte species had variable effects on host growth parameters, and this variation was often greater than variation between endophyte species (Figure 2). Plants inoculated with the A1 and S2 isolates did not differ by any recorded parameter. Plants inoculated with the S2 isolate had greater total, leaf, root biomass, and number of tillers than plants inoculated with the A2 isolate. While plants inoculated with the A1 isolate had increased height and number of tillers than S1 isolate and similar biomass as plants inoculated with the S1 and S2 isolates. Interestingly, plants inoculated with the S1 isolate had the greatest root: shoot ratio and the shortest height compared with plants inoculated with the three other isolates. The number of tillers was greater in plants inoculated with the S1 than with the A2 and S1 isolates.

### 3.7 Effects of the isolates within treatments

When comparing plants from the same population with different infection types within treatments, several interesting effects were observed (Table 5; Figure 4). For NC population plants, all infection groups had similar total, leaf, and root dry biomasses in each treatment combination (Table 5; Figure 4a,c). In the LWHN and LWLN treatments, plants infected with the S1 isolate had greater root:shoot ratio than plants with the A1 isolate. In the HWHN treatment, plants inoculated with S1 were shorter than uninfected plants, and plants infected with the S2 isolate had more tillers than uninfected plants (Table 5).

For the PA population plants, leaf dry biomass was lower for plants infected with the A2 and A1 isolates in the LWHN and LWLN treatments, respectively, in comparison to uninfected plants (Table 5; Figure 4b). Root dry biomass in plants with the A2 isolate was similar to uninfected plants but greater than plants inoculated with the A1 isolate in the HWLN treatment (Figure 4d). Total biomass of A1 and A2 infected plants in the LWHN treatment and A1 infected plants in the LWLN treatment was reduced compared to uninfected PA plants. In the HWLN treatment, root:shoot ratio of plants with A2 infection was greater than in A1 and E− plants. Height of A2 infected plants in the HWLN treatment was shorter than A1 and E− plants. Uninfected plants were also shorter than plants with A1 and A2 isolates in LWLN treatment. None of four isolates in PA plants had any effects on the tiller number at any treatment (Table 5).

## 4 DISCUSSION

The *E. alsodes* endophyte occurs commonly over a wide range of *P. alsodes* populations across the latitudinal gradient, whereas only a few populations in Pennsylvania host the other endophyte species, *E. schardlii* var. *pennsylvanica*. We found only one *P. alsodes* population where *E. schardlii* var. *pennsylvanica* was the sole endophyte. Such differences in the distributions of the two symbiotic endophyte species might be explained by selection from environmental factors. Plants that harbor beneficial microbial symbionts that increase resistance to biotic or abiotic environmental stresses may have higher fitness in a wider range of habitats, so frequency and range increases over time (Friesen et al., 2011; Reynolds et al., 2003). For example, *Rhizobium*, nitrogen‐fixing bacteria, associate with legume plant roots forming nodules that provide additional nitrogen nutrition to hosts. When soils are nitrogen poor as in overexploited farmlands in Zimbabwe, legumes may persist when other plants cannot and even increase soil fertility (Mapfumo et al., 2005). Bordeleau and Prévost (1994) emphasized that association with nitrogen‐fixing bacteria can allow persistence of legumes in the arctic where soils are nutrient poor and temperatures are extreme. Plant–mycorrhizal associations also can increase the frequency, persistence, and range of host plants (Klironomos, 2003; Smith & Read, 2010). However, the benefits of mycorrhizal associations depend on environmental conditions such as soil moisture, pH, temperature, and limiting nutrients, especially phosphorous (Bentivenga & Hetrick, 1992; Entry et al., 2002; Tuomi, Kytöviita, & Härdling, 2001). Mycorrhiza may also alleviate host stresses to various anthropogenic pollutants (Entry et al., 2002).

Asexual *Epichloë* are transmitted vertically and are not free‐living, so their frequency and distribution might be determined indirectly via selection by environmental factors on host plant fitness. If harboring the endophyte increases host fitness relative to uninfected plants across environments, then frequency and range of infected plants should increase with time (Clay, 1988, 1990). For example, Clay (1988) showed that the frequency of *E. coenophialum* in agronomic tall fescue increased in heavily grazed pastures over time because livestock avoided infected plants. If, alternatively, the cost of infection outweighs the benefit in certain environments, then infection frequencies should decrease relative to uninfected plants. For example, Novas et al. (2007) observed that in extremely harsh conditions in south Patagonia, *Epichloë* infection frequencies were reduced in several grass species. The same arguments apply to host grass species that harbor more than one *Epichloë* species. If infection by one endophyte species increases host plant fitness in certain environments relative to infection by another endophyte species, then we expect infection frequency and distribution to increase relative to plants infected with the other species or to uninfected plants. If natural selection is driving these differences in frequencies and distribution, then we also expect correlations of key environmental factors with the relative frequency of plants infected with different species of endophytes and uninfected plants. For example, Hamilton et al. (2009) determined that the frequency of a nonhybrid species of *Epichloë* from *Festuca arizonica* was positively associated with soil nutrients and heat load, whereas the frequency of a hybrid *Epichloë* species in the same grass was positively associated with soil moisture and pH.

Our study showed that the frequencies of the two endophyte species, *E. alsodes* and *E. schardlii* var. *pennsylvanica*, were also correlated with key environmental factors. Frequency of the widespread *E. alsodes* in the southern populations was associated with increased July Max temperatures (Tables 2 and 3). However, positive correlation with July precipitation may indicate that this endophyte may not mediate drought stress. This finding contrasts with previous experimental studies that showed that infection with an undetermined *Epichloë *sp. (but based upon its wide distribution, probably *E. alsodes*) from Indiana may increase drought resistance in *P. alsodes* (Kannadan & Rudgers, 2008).

The frequency of *E. alsodes* was also positively associated with soil nitrogen or organic matter (both variables are highly collinear). *E. alsodes* infected host plants may be associated with high nitrogen and phosphorous soils because of the increased nitrogen and phosphorous demand of producing high levels of NANL, a loline alkaloid. Alkaloids are nitrogen‐rich compounds and phosphorous is required in their synthesis (Faeth & Fagan, 2002; Schardl, Grossman, Nagabhyru, Faulkner, & Mallik, 2007). Alternatively, *Epichloë* infection itself may also enhance uptake of phosphorous from nutrient poor soils (Malinowski, Alloush, & Belesky, 2000). Increased phosphorous content in *Festuca rubra* plant tissues was demonstrated for *Epichloë festucae* infection (Zabalgogeazcoa, Ciudad, Vázquez de Aldana, & Criado, 2006).

In terms of biotic factors, *E. alsodes* infection frequency was negatively associated with insect damage. This negative association may reflect the powerful insecticidal effects of NANL, a loline alkaloid. NANL alkaloid concentrations produced by *E. alsodes* in *P. alsodes* plant tissues are high enough to cause larval and adult mortality for various insect species (Jensen, Popay, & Tapper, 2009; Popay, Tapper, & Podmore, 2009; Shymanovich et al., 2019).

Alternatively, in the few populations where *E. schardlii* var. *pennsylvanica* was detected, infection frequency was associated with soil nutrients but in opposite directions than for *E. alsodes* infected plants. Nitrogen and phosphorous were negatively correlated with *E. schardlii* var. *pennsylvanica* frequencies (Table 3). This negative correlation may indicate that infection by *E. schardlii* var. *pennsylvanica* allows *P. alsodes* to persist in marginal habitats where soil nutrients are low, possibly by facilitating nutrient uptake like some other *Epichloë* endophytes (Malinowski et al., 2000; Zabalgogeazcoa et al., 2006). However, this hypothesis is not supported by our greenhouse experiments. *E. schardlii* var. *pennsylvanica* infected plants did not grow better in the low nutrient treatments compared with *E. alsodes* infected, or with uninfected plants (Table 6; Figure 4a,c). Alternatively, the opposite direction of the correlation may be a statistical artifact because as the relative frequency of one endophyte species such as *E. alsodes* increases, the second endophyte frequency may decrease by default.

### 4.1 Overwintering survival

Our overwintering study provided some evidence that the widely distributed *E. alsodes* may be effective in enhancing host overwintering survival relative to plants with *E. schardlii* var. *pennsylvanica* or to uninfected plants. This finding also may be related to the negative correlation of *E. alsodes* infection frequencies with January Min temperatures (Table 1). However, this correlation was not statistically significant. Furthermore, we could not assess *E. alsodes* frequencies in more northern climates (Canada) where overwintering survival may be more critical. Chung, Miller, and Rudgers (2015) also found better survival of plants infected with unidentified *Epichloë* sp., (likely *E. alsodes* based on distribution and properties) in *P. alsodes* plants from Indiana compared to uninfected plants. Overwintering survival therefore remains a viable hypothesis for the widespread distribution and high frequencies of *E. alsodes*.

### 4.2 Transmission rates

Differences in transmission rates among *Epichloë* endophytes provide another explanation for differences in frequency and distribution that does not involve natural selection by the environment (Faeth & Sullivan, 2003; Ravel et al., 1997). *Epichloë* infection may be lost due to imperfect transmission (failure of hyphae to grow into seeds; Ravel et al., 1997), viability loss during seed storage, or randomly from adult plants (Afkhami & Rudgers, 2008; Cheplick & Faeth, 2009; Hill & Roach, 2009; Rolston et al., 1986; Siegel et al., 1985). Imperfect transmission can result in decreasing infection frequencies over time, even if endophytes increase fitness, if the rate of transmission failure is high (Ravel et al., 1997). Various *Epichloë* species in native grasses may have very different rates of transmission which could contribute to differences in frequency and range (Afkhami & Rudgers, 2008). However, the transmission rate hypothesis does not appear to explain differences in *E. alsodes* and *E. schardlii* var. *pennsylvanica* frequency and distribution, or the relative rarity of *E. schardlii*. Both species hosted by *P. alsodes* had high transmission rates (95%–100%) across all populations in our study. Chung et al. (2015) also detected high transmission rates in the populations in Indiana populations of *P. alsodes* infected with unspecified (but likely *E. alsodes*) *Epichloë* endophyte.

### 4.3 Compatibility

Similarly to other studies (e.g., Friesen et al., 2011; Oberhofer et al., 2014; Saikkonen et al., 2010), our inoculation trials provided additional evidence that plant genetic characteristics may control the compatibility with specific endophytes (Table 2). Plants from the North Carolinian population were similar in compatibility with both endophyte species. However, for the Pennsylvania population, inoculation success is strongly depended on the *Epichloë* species. Plants from the Pennsylvania population were more compatible with the widespread endophyte *E. alsodes* than *E. schardlii* var. *pennsylvanica* even though both species occur in these populations. It is unclear if the greater compatibility of *E. alsodes* compared to *E. schardlii* var. *pennsylvanica* is a cause or a result of its wider distribution and longer association with *P. alsodes*. *E. schardlii* var. *pennsylvanica* may have made a recent host jump from another co‐occurring grass, *Cinna arundinacea* (Ghimire et al., 2011) and this may partially explain the restrictive distribution of *E. schardlii* var. *pennsylvanica*.

Increased compatibility of host–endophyte genetic combinations may have improved host growth parameters. Several plant growth parameters indicated that resident host–endophyte combinations, which may be co‐adapted, were more beneficial to the host. For example, total biomass, leaf biomass (Figure 3), and tiller number were increased in North Carolina plants with the resident endophyte (A1), in the intermediate stress level treatments (HWLN, LWHN) compared to plants infected with the alien isolate (A2). Enhanced vegetative biomass and tiller number likely result in increased reproductive success and thus fitness (e.g., Faeth, 2009). Pennsylvania plants inoculated with the resident endophyte (A2) had increased root dry biomass and higher root: shoot ratio in several treatment groups compared to plants infected with the alien isolate (A1) (Figure 3). Greater root biomass may indicate better drought resistance and enhanced nutrient uptake (e.g., Malinowski & Belesky, 2000, Malinowski et al., 2000).

Host plant co‐adaptation with their residential endophytes may also depend on local environmental conditions. For example, the A1 isolate of *E. alsodes* that originated from the wettest habitat (Table 1) did not increase root biomass allocation in any plants. However, A2 isolate from the driest habitat (based on annual and July precipitation, Table 1) increased biomass allocation to roots in plants from both populations in intermediate stress level treatments (Figure 3) and thus may potentially increase host resistance to drought stress. Nevertheless, caution is necessary for two reasons. First, just a few isolates were tested in this study and, second, seeds from naturally uninfected genotypes were used for inoculations. Thus, additional inoculation experiments with other isolates and host plant populations and also with initially naturally infected genotypes may provide a stronger support for the hypothesis that plants and endophyte genotypes are co‐adapted.

### 4.4 Effects on host performance

Similar to other studies describing host–endophyte interactions as a mutualism–parasitism continuum (Junker, Draeger, & Schulz, 2012; Schulz & Boyle, 2005), our growth performance experiments with reciprocally inoculated plants from the NC and PA populations revealed the complexity of host and endophyte genotype and environment interactions on plant growth parameters. Different isolates from the same endophyte species may have different effects on plants from a given population. Moreover, effects of an endophyte on growth parameters were dependent on specific water‐nutrient conditions. In the resource‐rich treatment environment (HWHN treatment), infected plants did not differ much in growth parameters than uninfected plants, except height and tiller number (Table 5). Some differences in growth parameters between infected and uninfected plants, and between plants infected with different isolates, were detected when plants were grown in the moderately stressful treatments (HWLN and LWHN) or in some cases when in highly stressful environments (the LWLN treatment (Table 5; Figures 3 and 4)).

However, the major result of the performance experiment is that neither of two endophyte species or their isolates increased total plant biomass compared to uninfected plants, and in some cases, infection even reduced biomass (Figures 2 and 3) Chung et al. (2015) also found no effects of *Epichloë *sp. infection on total biomass of *P. alsodes* plants compared to uninfected plants. However, our experiment did show that endophytes had effects on the other growth parameters, including number of tillers, height, and root: shoot ratio. Infection with either species, but depending on isolate, may change tiller number compared to uninfected plants (Figure 2). For PA (Figure 2) and NC plants in the HWLN treatment (Figure 4), infection with A2 isolate increased root: shoot ratio which may increase drought resistance and nutrient uptake. NC plants infected with the S1 isolate of *E. schardlii* var. *pennsylvanica*, and PA plants with A2 isolate of *E. alsodes* showed reduced height in comparison to uninfected plants (Figure 2), which could be disadvantageous in woodland communities where light is reduced.

Our experiment also revealed interactions of plant population origin and endophyte isolates (Table 5). The effects of the *E. alsodes* isolates differed when introduced into plants from the North Carolina and Pennsylvania populations. For example, when infected with A2 isolate, NC plants showed only root biomass reductions, but the same isolate inoculated into PA plants showed reduced root and leaf biomass compared to uninfected plants from the same population (Figure 2). Root: shoot ratios increased for PA plants infected with the A2 isolate compared to uninfected plants but root: shoot ratios of NC plants infected with the same isolate did not differ from uninfected plants (Figure 2). Likewise, PA plants infected with A1 isolate had fewer tillers than uninfected plants, but tiller number of NC plants infected with the same isolate tended to be greater than in uninfected plants (Figure 2). Overall, our growth performance experiment showed complex outcomes of infection depending on endophyte species, isolate within species, population origin of the host plant and environmental factors. We did not find consistent or clear benefits of the endophyte infection by either species.

Our approach with artificial inoculations and a performance experiment with controlled water‐nutrient environments provided valuable results but had several limitations. First, because inoculations were made in naturally uninfected seedlings, we were not able to strictly control for plant genotypic variation within the population. These naturally uninfected plants may have once been infected with *Epichloë*, or may have been from plant lineages that had never been infected. Our inoculation and compatibility results suggest that plants infected by specific species and their isolates may be genetically distinct. Second, just a few plant and endophyte genotypes were tested for co‐adaptation. Third, our greenhouse experiment with potted plants in uniform potting soil, and controlled temperature, water, and nutrient conditions may or may not simulate natural environments. Fourth, we were unable to document seed production by plants infected with isolates of the endophyte species. None of the plants produced florets during the course of the experiment. Therefore, the growth parameters we measured are only assumed to affect reproduction and fitness. Fourth, we were unable to compare plant population effects for *E. schardlii* var. *pennsylvanica* because this endophyte was not successfully inoculated into PA plants.

## 5 CONCLUSIONS

Our study explored several explanations for the broader distribution range and higher frequency of the interspecific hybrid, *E. alsodes,* compared to the limited distribution of intraspecific hybrid species, *E. schardlii* var. *pennsylvanica*. Increased overwintering survival and better compatibility with a *P. alsodes* host from across the latitudinal gradient we sampled, may allow *E. alsodes* to persist over a broad latitudinal range. That the distribution and frequency of *E. alsodes* is correlated with maximum and minimum temperatures supports the overwintering success hypothesis. We did not find evidence that either endophyte species or their isolates provide consistent benefits in terms of growth parameters that would explain differences in distribution. However, our previous work (Shymanovich et al., 2017), showed that *E. alsodes* has another important benefit: production of loline alkaloids which may significantly reduce plant damage due to toxic effects on insect herbivores. *E. schardlii* var. *pennsylvanica* has insect deterrence properties, but does not have significant effects on insect survival and does not appear to produce alkaloids. Variation in insect defense mechanisms may be a key factor for variation in the distribution ranges. That *E. alsodes*, which produces high levels of NANL, a loline alkaloid that is nitrogen‐rich and may compete with plant functions for nitrogen, is positively associated with high‐nitrogen soils, suggests that the costs and benefits of alkaloid production may be important in dictating its distribution and frequency. Our overall results also support the more general hypothesis that interspecific hybridization provides greater genetic variation than intraspecific hybridization (e.g., Schardl & Craven, 2003) and thus greater potential for adaptation to wider range of, and more stressful, environments. Infection by the interspecific hybrid species, *E. alsodes*, appears to enable its host plant to persist across a wide variety of local environments across the 1,200 km latitudinal range that we sampled. In contrast, plants infected with the intraspecific hybrid species, *E. schardlii* var. *pennsylvanica*, appears restricted to a limited environments within this latitudinal range. Our correlational and experimental tests suggest that the broader range of *E. alsodes* infected grove bluegrass may be related to greater variation in alkaloid production and enhanced overwintering survival, as well as changes in some growth parameters. However, other hypotheses that do not involve natural selection by the environment, such as recent origination or host jump of *E. schardlii* var. *pennsylvanica* in Pennsylvania, or limited dispersal of *E. schardlii* var. *pennsylvanica*, cannot be excluded without further experimentation and observation.

## AUTHOR CONTRIBUTIONS

All authors contributed to the project design, data analyses, and writing of the manuscript. TS conceived the project.

## Data Availability

Data available in the Supplementary Material file.
