# Supplementary material for: Environmental factors affect the distribution of two Epichloë fungal endophyte species inhabiting a common host grove bluegrass (Poa alsodes)
Source: Ecol Evol. 2019 May 26;9(11):6624–42. doi: 10.1002/ece3.5241 (PMC6580270; doi:10.1002/ece3.5241)
Supplement: Supplementary file 1 [file ECE3-9-6624-s001.docx]

**SUPPORTING INFORMATION**

**TABLE S1.** Data on environmental factors and endophyte infections from *Poa alsodes* populations (South to North) for correlation analysis

| Population  Location | Infection *E. alsodes* | Infection *E. schardli*i var. *pennsylvanica* | January  Min,  ^o^C | July  Max, ^o^C | July  Precipi tation, mm | Annual  Precipita tion, mm | Organic  Matter, % | Estimated nitrogen release, lbs/a | Phos phorus, ppm | Potas sium, ppm | Magne sium, ppm | Cal cium, ppm | pH | Mean Insect  Damage, % |
| --- | --- | --- | --- | --- | --- | --- | --- | --- | --- | --- | --- | --- | --- | --- |
| NC-4 | 26 | 0 | -7 | 23 | 203.2 | 2012 | 4.6 | 136 | 49 | 66 | 52 | 283 | 4.7 | 9.8 |
| TN-3 | 100 | 0 | -4 | 30 | 147.8 | 1408.2 | 5.6 | 138 | 34 | 95 | 160 | 3892 | 7 | 3.1 |
| NC-2 | 96 | 0 | -2 | 30 | 149.6 | 1244 | 5.8 | 142 | 43 | 101 | 172 | 5881 | 7.7 | 2.4 |
| WV-6 | 90 | 0 | -11 | 27 | 122.4 | 1219.2 | 6 | 146 | 23 | 89 | 120 | 4669 | 7.6 | 1.4 |
| WV-5 | 100 | 0 | -9 | 25 | 122.9 | 1308.7 | 7.6 | 150 | 38 | 74 | 80 | 3837 | 6.4 | 2.5 |
| PA-19-L1 | 83 | 13.3 | -9 | 26 | 107.9 | 1099.7 | 2.1 | 87 | 9 | 26 | 40 | 254 | 4.6 | 1.1 |
| PA-19-L2 | 88 | 12.5 | -9 | 26 | 107.9 | 1099.7 | 4.8 | 132 | 11 | 28 | 89 | 1346 | 5.7 | 0.8 |
| PA-19-L4 | 100 | 0 | -9 | 26 | 107.9 | 1099.7 | 5.8 | 150 | 4 | 32 | 149 | 1587 | 6.3 | 0.7 |
| PA-8 | 50 | 46 | -10 | 27 | 125.5 | 1160.4 | 4.4 | 128 | 29 | 44 | 48 | 1071 | 6.1 | 8.5 |
| PA-9 | 100 | 0 | -10 | 27 | 116.8 | 1164.9 | 5 | 142 | 78 | 84 | 42 | 258 | 4.3 | 5.1 |
| PA-15 | 96 | 0 | -10 | 27 | 122.7 | 1121.9 | 5.8 | 150 | 18 | 58 | 114 | 630 | 5.4 | 2.7 |
| PA-18-L1&2 | 96 | 0 | -8 | 27 | 123.2 | 1198.6 | 6.8 | 150 | 13 | 52 | 141 | 606 | 5.3 | 1.6 |
| PA-18-L3 | 100 | 0 | -8 | 27 | 123.2 | 1198.6 | 5.5 | 150 | 26 | 65 | 75 | 1004 | 5.8 | 1 |
| PA-18-L4 | 0 | 100 | -8 | 27 | 123.2 | 1198.6 | 4.3 | 127 | 20 | 68 | 93 | 633 | 5.3 | 0.8 |
| PA-17 | 44 | 48 | -11 | 27 | 117.6 | 1190.2 | 7.4 | 150 | 11 | 60 | 543 | 2134 | 6.8 | 1.2 |
| PA-10 | 0 | 74 | -8 | 27 | 123.2 | 1198.6 | 5.7 | 150 | 22 | 103 | 95 | 743 | 4.7 | 2.8 |
| PA-16 | 96 | 0 | -11 | 27 | 110.7 | 1226.3 | 9.1 | 150 | 18 | 44 | 402 | 1315 | 6.3 | 1 |
| NY-11 | 100 | 0 | -10 | 27 | 122.7 | 1121.9 | 5 | 140 | 24 | 83 | 90 | 687 | 5.1 | 3.6 |
| NY-14 | 100 | 0 | -9 | 28 | 96 | 979.5 | 9.7 | 150 | 56 | 73 | 245 | 10707 | 7.5 | 7.1 |
| NY-13 | 98 | 0 | -13 | 27 | 105.7 | 1256.4 | 3 | 104 | 26 | 20 | 63 | 405 | 5.2 | 4.8 |
| NY-12 | 98 | 0 | -14 | 26 | 95 | 950 | 2.5 | 93 | 38 | 25 | 132 | 567 | 6.2 | 5.7 |
| MI-20 | 100 | 0 | -9 | 28 | 91.4 | 757 | n/a | n/a | n/a | n/a | n/a | n/a | n/a | n/a |

1. Populations MI20, PA18-L4, and NY11 were removed from correlation analyses for the latitudinal data set
2. Population PA19-1 and all not PA populations were removed from correlation analyses for the Pennsylvanian data set

**TABLE S2.** Source of uninfected *Poa alsodes* seeds and mycelial isolates from North Carolina (NC) and Pennsylvania (PA) populations used for artificial inoculations

| Origin of E- seeds | Endophyte/ Isolate from population-plant ID | Population of origin | Population natural infections frequencies | Coordinates | Elev.,m | Ann. prec., mm | July max Temp.,^o^C / Prec., mm |
| --- | --- | --- | --- | --- | --- | --- | --- |
| NC | *E. alsodes*/ NC-4-35 (A1) | Noland Divide Trail, Great Smoky National Park, NC | *E. alsodes* 24% | N 35^o^34.032' W 83^o^28.906' | 1815 | 2012 | 23^o^C/203 |
| PA | *E. alsodes*/ PA-17-24 (A2) | Elk State Park, PA | *E. alsodes* 44% *E. schardlii* var. *pennsylvanica* 48% | N 41^o^36.372' W 78^o^33.799' | 594 | 1190 | 27^o^C/118 |
| -* | *E. schardlii*/ PA-10-10 (S1) | Chapman State Park, PA | *E. schardlii* var. *pennsylvanica* 74% | N 41^o^44.915' W 79^o^10.368' | 456 | 1199 | 27^o^C/123 |
| PA | E. schardlii/ PA-17-44 (S2) | Elk State Park, PA | *E. alsodes* 44% *E. schardlii* var. *pennsylvanica* 48% | N 41^o^36.372' W 78^o^33.799' | 594 | 1190 | 27^o^C/118 |

*No seeds were used from this population. Only the fungal isolate of *E. schardlii* var. *pennsylvanica* was used in the experiment

**TABLE S3.** Vertical endophyte transmission with host, *Poa alsodes*, seeds

| Endophyte | Population | Population mean seed infection, % | Total seeds tested |
| --- | --- | --- | --- |
| *E. alsodes* | NC-2 | 98.61 | 72 |
| *E. alsodes* | TN-3 | 100 | 18 |
| *E. alsodes* | NC-4 | 100 | 72 |
| *E. alsodes* | WA-5 | 100 | 72 |
| *E. alsodes* | WA-6 | 100 | 72 |
| *E. alsodes* | PA-8 | 100 | 10 |
| *E. alsodes* | PA-9 | 100 | 72 |
| *E. alsodes* | NY-11 | 100 | 68 |
| *E. alsodes* | NY-12 | 100 | 72 |
| *E. alsodes* | NY-13 | 100 | 34 |
| *E. alsodes* | NY-14 | 100 | 72 |
| *E. alsodes* | PA-15 | 100 | 72 |
| *E. alsodes* | PA-16 | 100 | 72 |
| *E. alsodes* | PA-17 | 100 | 72 |
| *E. alsodes* | PA-18 | 100 | 72 |
| *E. alsodes* | PA-19 | 100 | 61 |
| *E. alsodes* | MI-20 | 98.61 | 72 |
| *E. schardlii* var. *pennsylvanica* | PA-8 | 100 | 72 |
| *E. schardlii*  var. *pennsylvanica* | PA-10 | 100 | 51 |
| *E. schardlii*  var. *pennsylvanica* | PA-17 | 100 | 72 |
| *E. schardlii*  var. *pennsylvanica* | PA-18 | 95.83 | 72 |
| *E. schardlii*  var. *pennsylvanica* | PA-19 | 100 | 39 |


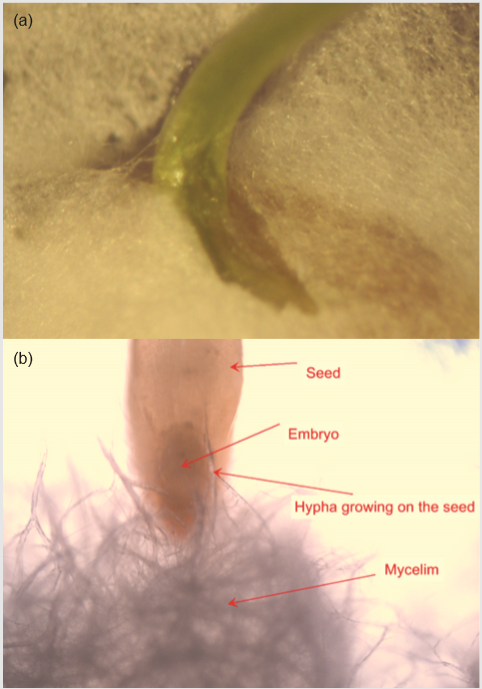


SUPPLEMENTARY FIGURE S1. Two endophyte inoculation procedures with seedling puncturing and insertion of a small portion of surrounding mycelium into a slit (a) and without puncturing when hyphae can grow into a seed and infect a developing seedling (b).


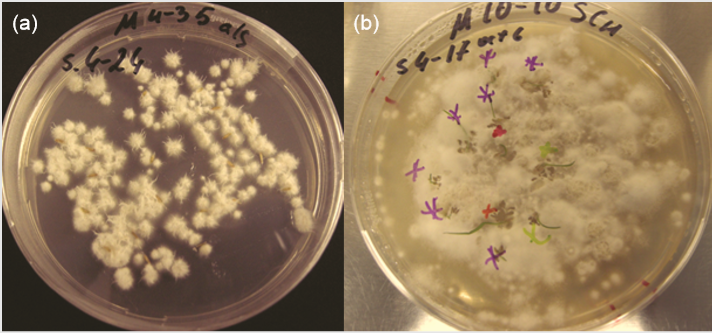


SUPPLEMENTARY FIGURE S2. Endophyte inoculation procedure for *Poa alsodes* seeds just placed on to 10-day-old mycelium (a) and for germinated seedlings inoculated with puncturing method (b).
